# Membership dropout rates and associated factors in a community-based health insurance scheme in southern Ethiopia: a mixed method study

**DOI:** 10.3389/frhs.2023.1203179

**Published:** 2023-11-28

**Authors:** Yosef Haile, Hanan Abdulkadir, Misgun Shewangizaw, Simeon Meskele, Kidus Temesgen, Temesgen Haile, Daniel Niguse, Samuel Hailegebreal, Getahun Gorfu Biratu

**Affiliations:** ^1^ Department of Public Health, School of Public Health, College of Medicine and Health Sciences, Arba Minch University, Arba Minch, Ethiopia; ^2^ Department of Biomedical Sciences, School of Medicine, College of Medicine and Health Sciences, Arba Minch University, Arba Minch, Ethiopia; ^3^ Hadiya Zone Health Department, Hossana, Ethiopia; ^4^ Department of Health Informatics, School of Public Health, College of Medicine and Health Sciences, Arba Minch University, Arba Minch, Ethiopia; ^5^ Department of Public Health, College of Medicine and Health Sciences, Wachamo University, Hossana, Ethiopia; ^6^ Outpatient Department, Sabian General Hospital, Dire Dawa, Ethiopia

**Keywords:** community-based health insurance, dropout rate, associated factors, Arba Minch, HDSS site

## Abstract

**Background:**

Dropout from community-based health insurance (CBHI) membership is a common problem in low-income countries, even if its implementation leads to substantial improvement in the utilization of essential health services. Few studies have addressed the factors contributing to dropout rates in southern Ethiopia. Therefore, the purpose of this study was to determine the rate of CBHI dropout in southern Ethiopia as well as any contributing factors.

**Methods:**

This mixed-method cross-sectional study was conducted among 460 randomly selected CBHI-enrolled households at the Arba Minch Health and Demography Surveillance System site from November 1, 2021, to April 30, 2022. The quantitative data were collected by an open data kit (ODK). using an interviewer-based structured questionnaire and analyzed using Statistical Package for the Social Sciences (SPSS) version 25.0. Multivariable logistic regression was applied to identify significant variables. The qualitative data were used to support the quantitative findings and were gathered through in-depth interviews (by the CBHI coordinator and three purposively selected health extension workers) and focus group discussions (in two randomly selected villages). The qualitative data were analyzed using thematic analysis. Finally, triangulation was used to present both the quantitative and qualitative findings.

**Results:**

This study found that 92 (21.5%) people stopped their community-based health insurance membership. The presence of sick adults [AOR = 0.281, 95% CI (0.136–0.581)], trust of participants in the contracted health facilities [AOR = 0.227, 95% CI (0.121–0.436)], and poor knowledge of the participants [AOR = 5.518, 95% CI (1.526–19.950)] were significant predictors.

**Conclusion:**

The magnitude of the dropout rate was high in this study when compared with the national target. The absence of a sick adult, the absence of trust among participants, and the poor knowledge status of the participants were significant predictors. We suggest that the health facility managers, the CBHI coordinating office, and the district health office give priority to implementing a wide range of knowledge improvement activities and a transparent system in public health facilities. Studies with longitudinal research designs are called for at a wide range of national levels to address the limitations of this study.

## Introduction

Community-based health insurance (CBHI) is a form of micro health insurance that attempts to protect against the cost of disease and enhance access to high-quality healthcare for low and middle-income rural households. The program typically includes community members pooling money to lower healthcare costs, and it is voluntary (1–3).

Globally, about 2 billion people are facing catastrophic or impoverishing health spending, even if there has been an improvement in health service coverage after the global commitment to achieve universal health coverage by 2030. This problem is mainly caused by' high out-of-pocket payments when visiting health facilities to obtain the needed essential health services (4–6). As the WHO reported, one of the two elements of universal coverage is access to efficient preventive and curative interventions, and the other is protection against financial hardship because of using services (7).

In Ethiopia, in terms of mitigating financial risk, the National Health Account (NHA)-8 reports that, in 2019–20, out-of-pocket (OOP) health spending accounted for 30.5% of total health expenditure (THE), with 4.2% of Ethiopian families experiencing catastrophic medical costs (3). Ethiopia is dedicated to achieving universal health coverage (UHC) through increasing access to high-quality medical care that is available, cheap, acceptable, and convenient for all households. Because financial risk protection is a crucial element of UHC, Ethiopia has started introducing comprehensive and long-lasting financial risk protection through a health insurance program known as community-based health insurance (8–10).

Even though there were many challenges in the implementation of CBHI for healthcare financing in many low- and middle-income countries, it brought substantial improvements in the utilization of essential health services. In Ethiopia, CBHI-affiliated health facilities saw an increase in yearly outpatient visits of 111% and in income of 47%. There was an 11% rise in patient satisfaction. These findings and the comparatively high CBHI membership rates indicate that the Ethiopian CBHI has been able to overcome the primary obstacle—poor service quality—that has dogged other Sub-Saharan African CBHI programs (8, 11–14).

Despite these substantial improvements in the health sector in Ethiopia, dropout from CBHI membership, especially among the first members of the pilot study, is high (15). According to the Arba Minch Zuria district health office report (study area), CBHI implementation was started in 2016 G.C. in the district for the first time in the Gamo zone (after the pilot or first phase of implementation of CBHI was completed in Ethiopia). Compared to the starting point in 2016, more than 20% of the initially enrolled households had dropped out of CBHI by 2020 (16).

Dropout from CBHI membership could be affected by individual, household, community, and system-level factors such as age, educational level, family size, trust in the health institution, trust in the program managers, poor perceived service quality, providers' attitude, benefits package, culture, community involvement in scheme development, governance, financial, and delivery arrangements (15, 17–19).

In Ethiopia, three studies were carried out on this specific topic; however, two of them were conducted during the first phase of implementation in the pilot districts (15, 20). After the high enrollment of members in the second phase of implementation, only one study was conducted in Gonder, northern Ethiopia. According to this study, a high number of members dropped out of the CBHI membership (19). The current study was conducted in southern Ethiopia because there are socioeconomic and demographic disparities between northern and southern Ethiopia. In addition, the current study applied a mixed-methods approach involving qualitative data in addition to quantitative data to explore the reasons for dropout from CBHI that were not explored by previous studies. Therefore, this study provides updated information regarding the dropout rate from the CBHI scheme in the southern part of Ethiopia as well as identifies any contributing factors.

## Methods and materials

### Study area and period

The Arba Minch Health and Demographic Surveillance System (AM-HDSS) sites, which cover the nine villages in the Arba Minch Zuria district, were used for this study. The administrative center of the district, Arba Minch Town, is 505 kilometers (km) south of Addis Ababa, the nation's capital city. Around 195,858 people were living in the district as a whole in 2017 (21). The district had seven health centers and 26 health posts. According to the report of the CBHI coordinating office of Arba Minch Zuria district health office, Arba Minch Zuria district is one of the first districts to start CBHI implementation in the Zone in 2009 E.C (16). The data was collected from Nov 1, 2021, to April 30, 2022.

### Study design

A community-based cross-sectional study design employing both quantitative and qualitative methods was used.

### Source population

The source population was all household heads that were enrolled in the CBHI scheme.

### Inclusion and exclusion criteria

All household heads that have ever registered for CBHI on the HDSS site were included in this study; however, those household heads who had only been CBHI members for less than one year were not.

### Sample size determination and procedure

#### Sample size determination

Using a single population proportion formula, the sample size was calculated while taking the following presumptions into account: 95% confidence level (1.96), P0 = 31.7% proportion of dropouts from the study conducted in Manna district of Jimma Zone (10), *d* = 5% margin of error,

$$n=\frac{\left(Z1/2)2p(1-p\right)}{d2}$$

$$\begin{array}{rl} n= & \frac{\left(1.96)2\;0.32(1-.32\right)}{(0.045)2}\\ = & \;418 \end{array}$$

By adding a 10% non-response rate, the final sample size was determined to be 460.

#### Sampling procedure

The nine villages in the Arba Minch Health and Demography Surveillance Site (HDSS) were selected purposefully. The sample size was then proportionally allocated to each of the villages according to the number of enrolled households in each. Finally, a simple random sampling technique was used to choose the households. The district CBHI scheme coordination office provided the lists of registered household heads for the CBHI program. Together with the leaders of the villages and the women's development army, their usual residence was determined.

### Data collection tool and technique

#### For quantitative data collection

An interviewer-based structured questionnaire that was adapted from different related literature was used to collect relevant information (16, 19, 20). The questionnaire included questions about socioeconomic status, demographics, health status, and factors related to healthcare facilities, as well as questions about research participants' prior knowledge of and expectations for CBHI and other specific CBHI-related issues. Nine well-trained data collectors conducted in-person interviews with the pre-tested, structured questionnaire to gather data.

Two trained supervisors who were MPHs in public health were hired. The supervisors and data collectors received training on how to use the data collection technology, conduct interviews, use specific questioning strategies, and protect respondents' privacy and confidentiality.

#### For qualitative data collection

The qualitative information was gathered using a focus group discussion (FGD) and an extensive interviewing guide. The data gatherers were fluent speakers of the local language and masters of public health with experience in qualitative data collection. The district CBHI coordinator and three purposively selected health extension workers (HEWs) were interviewed as key informants. Key informant interviews were conducted at the offices and lasted 30–35 min. The interviews were audio-recorded. Two focus group discussions (FGDs) were held among CBHI members who hadn't renewed their cards for more than one year in two randomly selected villages (with a group of 7–11 participants). The FGD discussions were also audio-recorded.

### Study variable

#### Dependent variable

Dropout from community-based health insurance was the dependent variable.

#### Independent variables

Socio-economic and demographic factors, including household head's age, sex, occupation, educational status, marital status, household size, wealth index, health status, and health facility-related factors (self-perceived health status, presence of chronic illness, sick adults, and sick under-five children in the last 3 months, time taken to reach HFs, and waiting time), Experience and expectation of study participants on CBHI (CBHI-related meetings and training, affordability of premiums, length of enrollment, trust in the CBHI scheme, trust in health professionals, and trust in health facilities), as well as other individual CBHI-related factors such as attitude toward CBHI and knowledge of study participants on the concept of CBHI, were independent study variables.

### Operational definition

#### Dropout from CBHI

Household heads who were members of the CBHI scheme for more than one year but were not enrolled at the time of the survey were classified as dropouts. It was answered by observing the CBHI identification card.

#### Renew

Household heads who were members of CBHI for more than 1 year and who were still enrolled in or had renewed CBHI identification cards at the time of the survey were considered “renewed”.

#### Knowledge about CBHI

Households were asked seven sets of items about CBHI, and then responses were classified into three categories: high, medium, and low knowledge. Accordingly, those who scored 14, between 12 and 13, below 12 points were considered highly knowledgeable, medium knowledgeable, and low knowledgeable, respectively.

#### Attitude towards CBHI scheme

Participants were given a list of five questions, each of which was graded on a Likert scale with five possible responses ranging from strongly disagree to strongly agree. The overall score, which reflects the respondent's place along the scale of favorableness toward CBHI, was examined under the supposition of summated scales. As a result, the possible cumulative score for five items ranges from a minimum of 5 to a maximum of 25. The most favorable attitude is demonstrated when each person's overall score is close to 25, and the most unfavorable attitude is demonstrated when the score is close to 5. Accordingly, it was divided into two categories based on this favorableness continuum: unfavorable attitudes, which scored below the median, and favorable attitudes, which scored above the median.

#### Household wealth index

Based on the types of consumer items owned by households, asset data was gathered, factor scores were generated using PCA, and the composite scores were then divided into five quantiles. The poorest 20% quantile was identified as the first 20% quantile, and the richest 20% quantile was identified as the last 20% quantile.

#### Trust in healthcare facilities

This item was asked, “Have you trusted the contracted health facility?” Those who responded “yes” were considered trusted in the health facilities; otherwise, they were not trusted.

#### Trust in health professionals

This item was asked, “Have you trusted the health professionals in health facilities? Those who responded “yes” were considered trusted by the health professional; otherwise, they were not trusted.

#### Health facilities

According to this study, health facilities include health centers and hospitals serving the population of the study area.

### Data quality assurance

Various actions were taken to maintain data quality assurance. Data collectors and supervisors received a two-day training on the technique before data collection about overviews regarding CBHI dropout, each component of the tool, and ethical issues in order to ensure the quality of the data. The principal investigator and supervisor regularly verified the acquired data for accuracy and completeness. For better comprehension by the data collectors and respondents, the English version of the questionnaire was translated into the local language. To ensure consistency, the questionnaires were then reverse-translated into English. Using 5% of the overall sample size, the data collection tools were pretested at Lante village in the Arba Minch Zuria District. As a result of the pretest, any flaws in the design of the research instruments were restructured, and they were strengthened in terms of their clarity, understandability, and ease of use for gathering the necessary data for the study.

### Data processing and analysis

#### Quantitative data analysis

Data were gathered using an open data kit (ODK) and then exported to SPSS version 25 for analysis. Descriptive statistics were done. Both binary and multivariable logistic regression were applied. Candidates for the multivariable logistic regression model included variables from bivariate analysis with a *p*-value of less than 0.25. The Hosmer and Lemeshow goodness of fit test was used to determine the fitness of the model. With a 95% confidence interval and a *p*-value of 0.05, statistical significance was declared. The outcome was then displayed in text, charts, percentages, tabulations, and textual data.

#### Qualitative data analysis

Thematic analysis was used to transcribe, quote, code, and analyze the data. Themes and categories emerged from the text data after repeated reading. The focused group discussion (FGD) and in-depth interview were all designed with barriers to CBHI adherence in mind. A coding system that indicated common topics seen in the transcript review was established based on this theme. During the data analysis period, the codes were redefined. As a result of this process, descriptive categories were produced to show the components, which were then given names to reflect the foundation of an explanation for the phenomenon being observed. Triangulation was used to present both the quantitative and qualitative data outputs. The quotations that served as the best explanations for the quantitative results were chosen and listed under each quantitative finding to strengthen the quantitative findings.

## Results

### Socio-demographic characteristics of study participants

Of the 460 CBHI users selected to be enrolled in this study, 428 of them responded to the interview, giving a response rate of 93.04%. The mean age of the study participants was 45.18 (SD ± 10.85). In the majority (369, or 86.2%) of households, the male was the household head, and most of them (403, or 94.2%) were married. Most of the study participants (313, or 73.1%) were protestant religion followers, followed by Orthodox religion followers (110, or 25.7%). Regarding educational status, most of the study participants (207, or 48.4%) were unable to read or write. Farmers were dominant in magnitude from the occupation categories 335 (78.3%), followed by housewives 46 (10.7%). In most of the households, the number of members was more than or equal to 5,342 (79.9%). Almost half of the households (219, 51.2%) had under-five children in their house (Table 1).

**Table 1:** Socio-demographic characteristics of study participants in Arba Minch HDSS site, Ethiopia, 2022 (*n* = 428).

Variables	Categories	Frequency	Percent, %
Sex	Male	369	86.2
	Female	59	13.8
Age	20–29	13	3.0
	30–39	116	27.1
	40–49	168	39.3
	≥50	131	30.6
Marital status	Single	2	0.5
	Married	403	94.2
	Divorced	5	1.2
	Widowed	18	4.2
Religion	Orthodox	110	25.7
	Protestant	313	73.1
	Muslim	2	0.5
	Other	3	0.7
Level of education	Unable to read and write	207	48.4
	Primary education	171	39.9
	Secondary education and above	50	11.7
Occupation	Farmer	335	78.3
	Housewife	46	10.7
	Merchant	25	5.8
	Daily laborer	16	3.7
	Pretty trader and other	6	1.4
Household Size	<5 members	86	20.1
	More than or equal to 5 members	342	79.9
Age above 65 years in the household	Yes	59	13.8
	No	369	86.2
Presence of under-five children	Yes	219	51.2
	No	209	48.8
Wealth status	Poor	85	19.9
	Lower middle	88	20.5
	Middle	89	20.8
	Upper middle	48	11.2
	Richest	118	27.6

### Knowledge of study participants about CBHI

**Figure 1:**
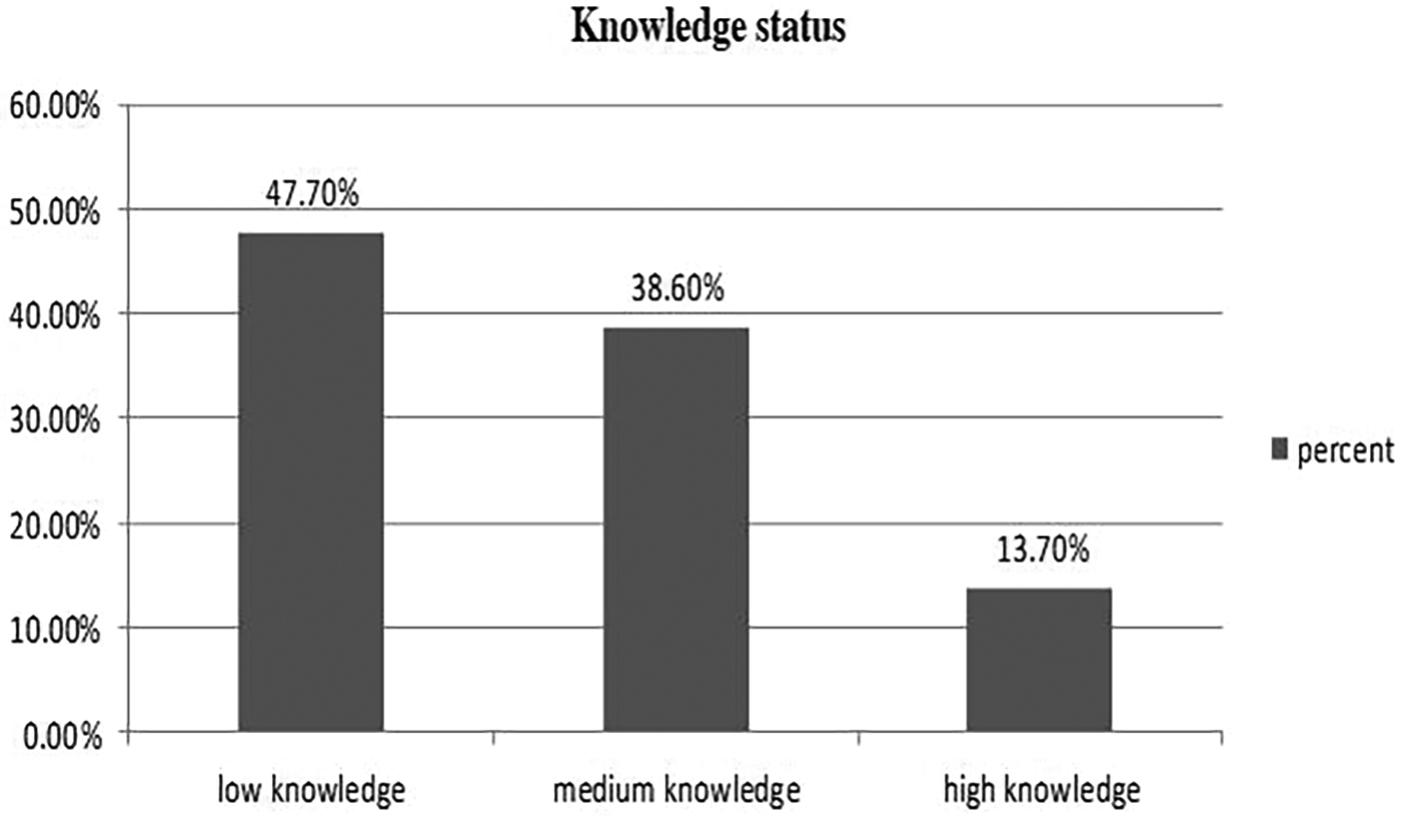
Knowledge status of the study participants about CBHI (*n *= 428).

Only 59 respondents (13.8%) in this study had high knowledge about CBHI, 204 (47.7%) had low knowledge, and the remaining respondents had medium knowledge (Figure 1).

### The attitude of study participants toward CBHI

**Figure 2:**
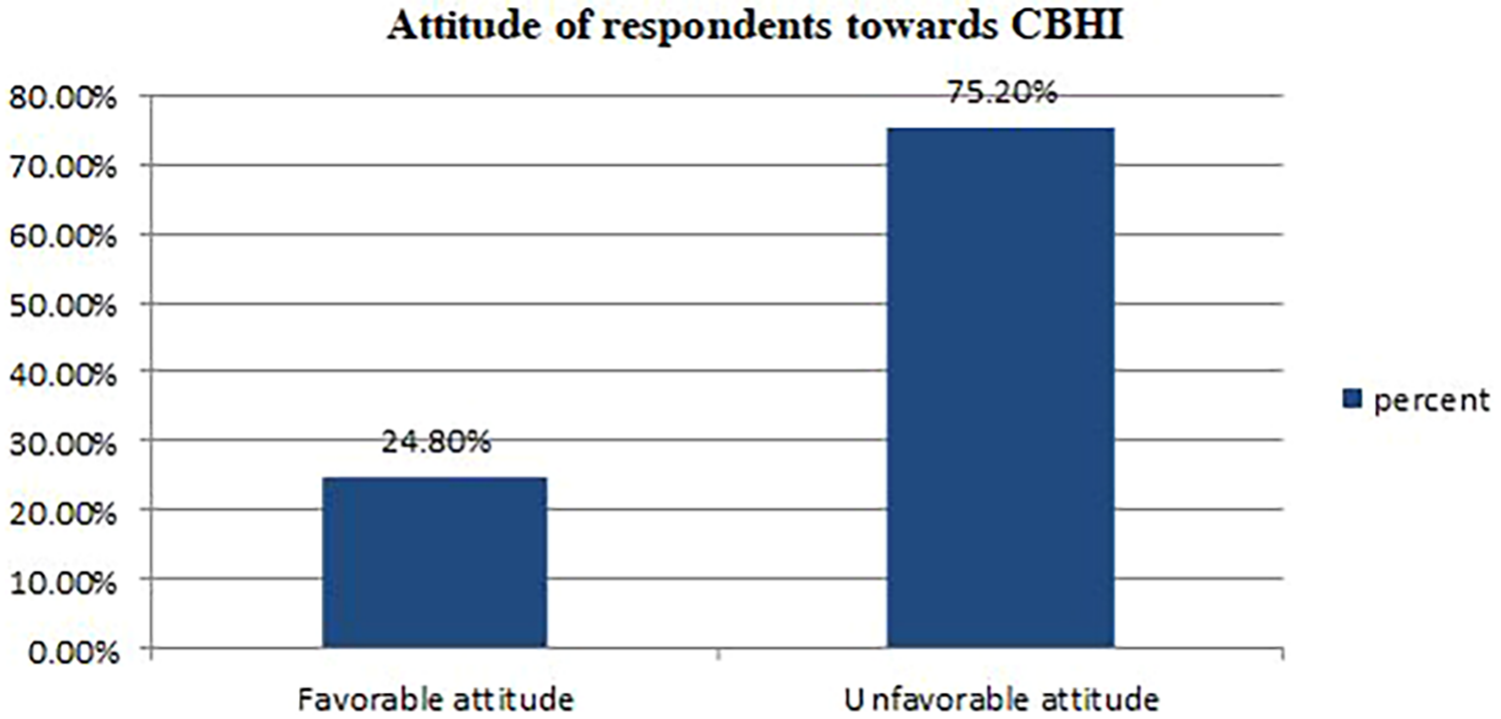
Attitude status of the study participants towards CBHI (*n *= 428).

More than three-fourths (75.2%) of study participants scored less than or equal to the median on attitude-related questions; therefore, they were categorized as CBHI scheme members who had an unfavorable attitude toward CBHI (Figure 2). It was supported by the qualitative findings as follows:

*“I was a member of the CBHI scheme, but currently, I am not a member of CBHI because I didn’t get any benefit from it, and I do not support others being members of CBHI. …. When I visited the health facility a year ago, I was dissatisfied with the services they provided for me because health providers provided only beds for me; almost all drugs were bought from private drug stores. Starting from that time, I am not happy with this CBHI program’’ (one of the FGD participants).*

### Presence of sick adults in their house

From the total study participants, around 126 (29.4%) adults were ill in the last 3 months before actual data collection.

### Trusts of study participants in health facilities

Of the total study participants, more than half (227, or 53%) have not trusted the health facilities. The qualitative finding supports this finding as follows: *“Most of the members of CBHI have no trust in the health facilities as well as health professionals because they are not getting recommended health services… As I observed, there are insufficient drugs and other services like laboratory services in most of the health facilities; this makes them dissatisfied and not trust the health facilities”(one of the key informants)*.

### The magnitude and reasons for dropout from CBHI membership

**Figure 3:**
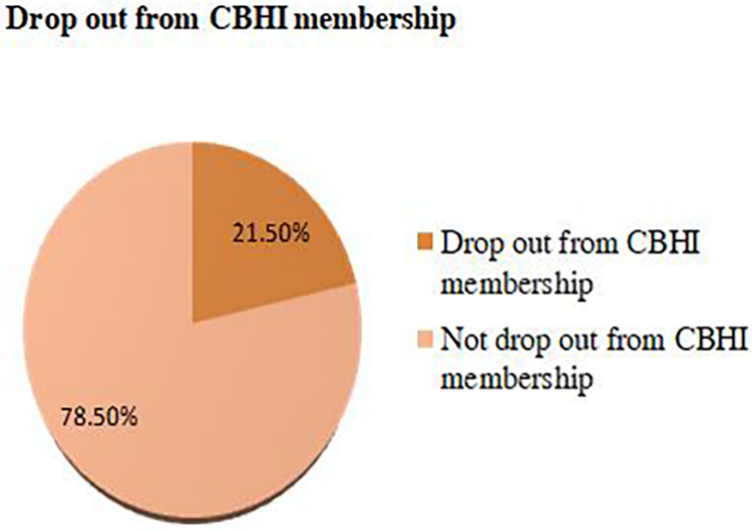
Drop out from community-based health insurance scheme in Arba Minch HDSS site, 2022 (*n *= 428).

**Figure 4:**
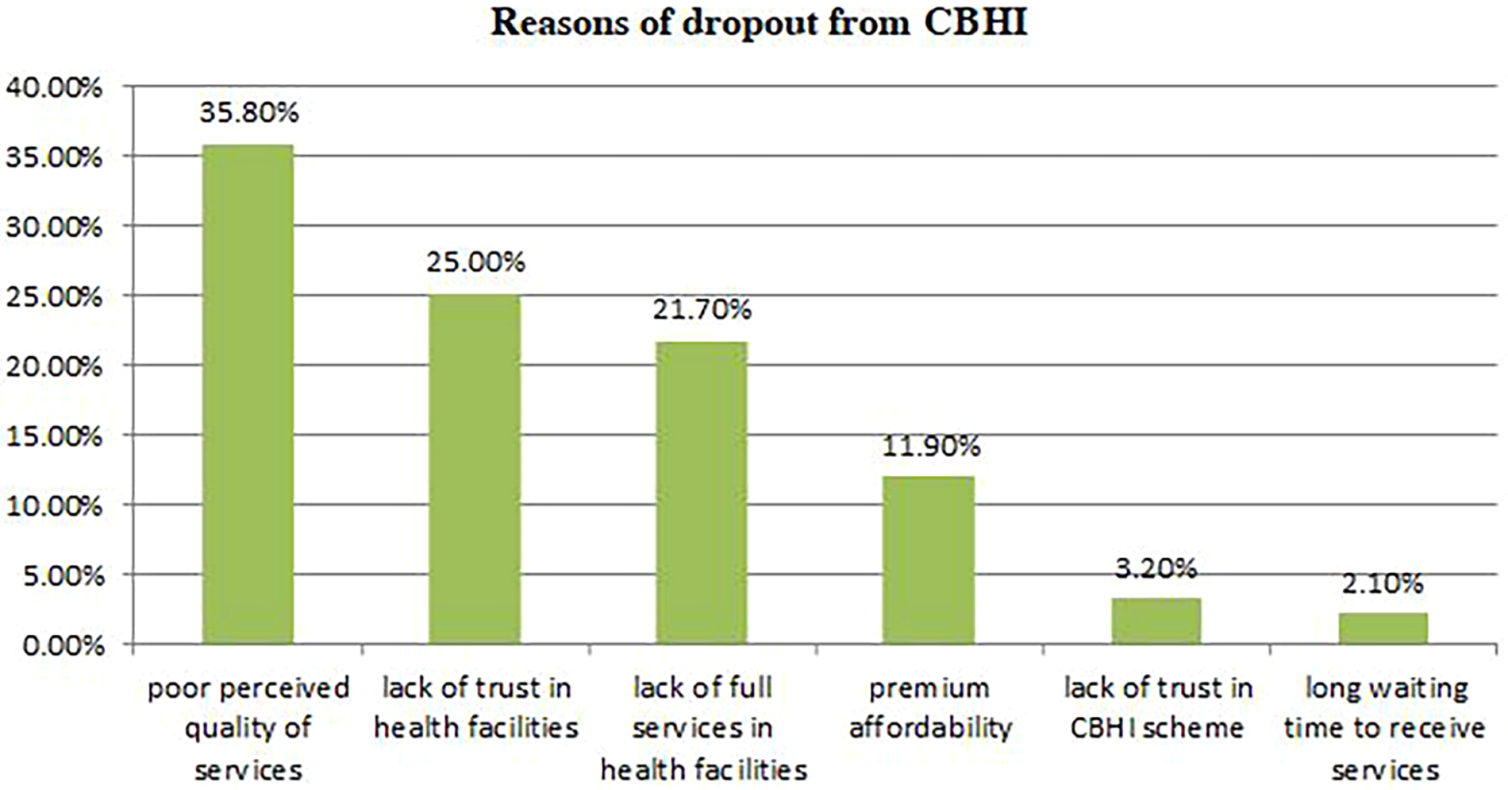
The reasons for dropout from community-based health insurance.

According to this study, the dropout rate from community-based health insurance membership was 92 (21.5%) (Figure 3). In most of the cases, the reasons behind dropout from CBHI were poor perceived quality of service 33 (35.8%), lack of trust in health facilities 23 (25.0%), lack of full services in health facilities 20 (21.7%), premium unaffordability 11 (11.9%), lack of trust in CBHI scheme 3 (3.2%), and long waiting time to receive services 2 (2.1%) (Figure 4). This finding was supported by the qualitative findings as follows: “*In the past, there was a gap in awareness about the importance of CBHI among communities, but this is not a problem currently because all people have awareness about the benefits of CBHI. At this time, the reasons that make members drop out of CBHI are inadequate drug and laboratory reagent supply in health facilities, poor quality of health services, limited availability of health services, and attitudinal problems among health providers for CBHI members* (one of the key informants).

### Factors associated with dropout from community-based health insurance

All the variables that had an association at a significance level of less than 0.20 with dropout from CBHI in the bivariate analysis, namely, household size, knowledge status, CBHI enrollment length, trust in the CBHI scheme, trust in the health facilities, and having sick adults in their house, were entered into a stepwise backward multivariable logistic regression analysis. After controlling for the effect of other predictor variables in the final model of multivariable logistic regression, the presence of sick adults in their households, trust of participants in the health facilities, and knowledge status on CBHI were significantly associated with dropout from the CBHI scheme with a *p*-value of <0.05. Household heads having a sick adult in their households in the previous 3 months of data collection had a 78% reduced chance of dropout from the CBHI scheme than those having no sick adult in their households [AOR = 0.281, 95% CI (0.136–0.581)]. The trust of participants in health facilities was also another predictor of dropout from CBHI in our study. Those who had trust in the health facilities they were using had a 77.3% reduced chance of dropping out of community-based health insurance schemes [AOR = 0.227, 95% CI (0.121–0.436)]. Further, household heads with low knowledge of the concept of CBHI were about 5.5 times more likely to drop out of CBHI than those who had low knowledge of the concept of CBHI [AOR = 5.518, 95% CI (1.526–19.950)] (Table 2).

**Table 2:** Multivariable logistic analysis for factors independently associated with dropout from community-based health insurance and associated factors in Arba Minch HDSS site (*n* = 428).

Variables		Dropout status		COR	AOR [95% CI]	*p*-value
		No dropout (%)	Dropout (%)			
Sick adult in their house	Yes	120 (92.3)	10 (7.7)	0.222	0.281 [0.136–0.581]	0.001
	No	216 (72.5)	82 (22.5)	1	1	
Trust in Health facilities	Yes	186 (92.5)	15 (7.5)	0.157	0.227 [0.121 –0.436]	0.000
	No	150 (66.1)	77 (33.9)	1	1	
CBHI enrollment length	1–2 years	173 (74.6)	59 (25.4)	1.685	1.693 [0.884–2.883]	0.053
	More than 2 years	163 (83.2)	33 (16.8)	1	1	
Knowledge	Low	155 (76.0)	49 (24.0)	5.93	4.37 [1.23–15.55]	0.022
	Medium	125 (75.8)	40 (24.2)	5.98	5.51 [1.52–19.95]	0.009
	High	56 (94.9)	3(5.1)	1	1	

Where, 1 = Reference group, Hosmer and Lemeshow: *p* = 0.93 classification power = 78.5% Nagelkerke *R* square = 0.24.

## Discussion

This community-based study attempted to determine the magnitude of dropout from community-based health insurance and its associated factors in southern Ethiopia. According to this study, the overall dropout rate from community-based health insurance was 92 (21.5%), with a 95% confidence interval of 17.5–25.7. This finding was in line with study findings conducted among pilot districts in Ethiopia (25%) and Uganda (25.1%). However, the finding was lower when compared to the study conducted in the Jimma zone (31.9), two districts of North East Ethiopia (29.14%), and Dera district, North West Ethiopia (37.3%) (15, 19, 20). The sociodemographic factors, including wealth status, educational status, cultural background, and others, may vary in these areas, which could account for this discrepancy. Length of enrollment may be another possible reason because the above studies were conducted in the pilot districts, which may affect the adherence level of community-based health insurance. As the length of the enrollment increases, the dropout rate may increase.

The reasons for dropout from community-based health insurance were poor perceived quality of service, lack of trust in health facilities, lack of full services in health facilities, premium unaffordability, lack of trust in the CBHI scheme, and long waiting times to receive services. The qualitative finding also supports this finding, as stated in the result part. The findings of the studies conducted in the Dera district, North West Ethiopia, and the Manna district of Jimma Zone partially support these findings (15, 19).

According to the findings of this study, the presence of adult sick patients in their houses in the last three months is one of the significant factors affecting the dropout rate from community-based health insurance schemes. Those household heads having sick adult patients in the previous three months in their house were less likely to drop out of community-based health insurance than those households having no sick adult in their house. This could be due to the fact that those household heads who have sick adults in their house might be worried about the cost of health services. This indicated that there is a high linkage between households with chronic diseases and adherence to community health insurance.

The other significant predictor of low adherence or dropout from community-based health insurance was the trust of study participants in the health facilities they were visiting. Those household heads with low trust in the contracted health facilities were more likely to be dropped from community-based health insurance than their counterparts. A possible explanation might be that the study participants may not be satisfied with the services provided for them at the time of illness in the contracted health facility. The health facilities must provide quality health services for the clients; otherwise, poor quality of services may decrease the trust of members of community-based health insurance in the CBHI-affiliated health facilities, and furthermore, this may increase the dropout rate of the members of CBHI. The study results from the Manna district, one of the Jimma zone's pilot districts, provided support for this finding (15).

In addition, the knowledge of study participants was a significant predictor of the dropout rate from the CBHI scheme. Those household heads with low knowledge regarding CBHI were more likely to drop out of the CBHI scheme than those with high knowledge of the CBHI concepts. The findings of a study conducted in Ethiopia support this finding (19). However, this finding was not supported by the findings of the study conducted in Rwanda, which revealed that there is no linear association between knowledge of the CBHI scheme and dropout from CBHI (17, 20).

### Limitations of this study

The cross-sectional data in this study, which lacks cause-and-effect information, may be one of its shortcomings. This study may also have recall bias since it depends on the verbal responses of study participants. In addressing this form of bias, we provided questions with an opportunity to remember it. A social desirability bias could also exist in this study. We have also consulted a variety of information sources to reduce this bias.

## Conclusion

The magnitude of the dropout rate was high in this study when compared with the national target. The absence of a sick adult in their home, the absence of trust among participants, and the poor knowledge status of the participants on the concept of CBHI were significant predictors. We suggest the health facility managers, the CBHI coordinating office, and the district health office give priority to implementing a wide range of knowledge improvement activities and a transparent system in public health facilities. Studies with longitudinal research designs are called for at a wide range of national levels to address the limitations of this study.

## Data Availability

The raw data supporting the conclusions of this article will be made available by the authors, without undue reservation.
